# Hidden burden of malaria in pregnancy in hard-to-reach areas of Odisha, India: challenges to elimination and the case for integrating molecular surveillance

**DOI:** 10.3389/fpubh.2026.1862569

**Published:** 2026-08-18

**Authors:** Sonali Sandeepta, Ramakanta Rana, Jyoti Ghosal, Arundhuti Das, Debarpita Mohanty, Biswadeep Das, Manoranjan Ranjit, Shubhashisha Mohanty, Debakanta Sandhibigraha, Sanghamitra Pati, Madhusmita Bal

**Affiliations:** 1 Indian Council of Medical Research-National Institute of Health Research, Bhubaneswar, Odisha, India; 2 School of Biotechnology, Kalinga Institute of Industrial Technology (KIIT) University, Bhubaneswar, Odisha, India; 3 Directorate of Public Health, Bhubaneswar, Odisha, India

**Keywords:** anemia, asymptomatic infection, malaria elimination, malaria in pregnancy, molecular surveillance, public health, sub-patent malaria

## Abstract

**Background:**

Malaria in pregnancy (MiP) remains a major public health concern due to its detrimental effects on maternal and foetal health, posing a substantial challenge to malaria control and elimination efforts. Asymptomatic/Sub-patent malaria infections, which escape detection under routine surveillance warrant focused investigation in high-burden, hard-to-reach areas of Odisha, India.

**Methods:**

Pregnant women were enrolled as part of repeated cross-sectional community-based *DAMaN Assessment* surveys conducted in 2019, 2021, and 2024 in malaria-endemic districts of Odisha. Malaria infection was detected using rapid diagnostic tests (RDTs), microscopy, and polymerase chain reaction (PCR), and hemoglobin levels were measured using a HemoCue Hb 201 + analyser. Prevalence estimates were calculated with 95% confidence intervals (CIs). Multivariable logistic regression was used to identify factors associated with malaria infection and sub-patent malaria, and to examine the association between malaria infection and anemia.

**Results:**

A total of 861 pregnant women were included. Overall malaria prevalence was 33.0% (95% CI: 29.9–36.3), varying from 51.1% (95% CI: 45.8–56.5) in 2019 to 9.8% (95% CI: 7.0–13.6) in 2021 and 41.1% (95% CI: 33.8–48.8) in 2024. Among malaria-positive women, 79.6% were asymptomatic and 74.3% (95% CI: 68.7–79.2) had sub-patent infections. Residence in a high-transmission season was independently associated with malaria infection (adjusted odds ratio [aOR] 8.23, 95% CI: 4.30–15.78), whereas Indoor residual spraying (IRS) (aOR 0.02, 95% CI: 0.01–0.04) and bed-net use (aOR 0.25, 95% CI: 0.10–0.62) were protective. Among malaria-positive women, increasing maternal age (aOR 1.05, 95% CI: 1.01–1.15) and higher gravida (aOR 1.70, 95% CI: 1.07–1.25) were associated with sub-patent malaria. Overall anemia prevalence was 62.3% (95% CI: 58.9–65.5), but malaria infection was not significantly associated with anemia after adjustment for confounders (aOR 0.98, 95% CI: 0.55–1.74).

**Conclusion:**

The high burden of asymptomatic/sub-patent malaria, coupled with mixed *Plasmodium* infections and anemia among pregnant women, represents a significant impediment to malaria elimination in endemic, underserved regions. These findings underscore the need for strengthening pregnancy-focused active surveillance in high burden settings using sensitive molecular tools in sentinel surveillance sites to guide targeted intervention strategies within programmatic frameworks to support India’s goal of malaria elimination by 2030.

## Introduction

Malaria, caused by *Plasmodium* species, remains one of the major global health problems, particularly in tropical and subtropical regions despite considerable progress in control efforts (1). In India, substantial gains have been achieved, with a marked reduction in malaria incidence and mortality between 2017 and 2023, leading to the country’s official exit from World Health Organization’s High Burden to High Impact list in 2024 (2). However, the malaria burden remains highly heterogeneous within India, with certain states and districts still contributing disproportionately to malaria cases and deaths (3).

Odisha, a coastal state along the Bay of Bengal comprising approximately 4% of India’s population, consistently occupies a prominent position on the national malaria epidemiological map, accounting for more than one-quarter of the reported malaria cases in India (4). Despite a substantial reduction in malaria positivity achieved by 2018 following the implementation of the large-scale state-led malaria elimination initiative, Durgama Anchalare Malaria Nirakaran (DAMaN), in conjunction with the National Malaria Control Programme, Odisha experienced a marked resurgence in malaria positivity, reaching approximately 63% in 2024 (5). The extensive forest cover, hilly and inaccessible terrain, and humid climatic conditions in the state are recognized as ecological determinants facilitating sustained vector proliferation and endemic malaria transmission (4). However, the contribution of asymptomatic and sub-patent malaria infections to the maintenance of persistent transmission cannot be excluded, particularly given the limited sensitivity of routine microscopy and rapid diagnostic tests, the substantial genetic diversity of circulating parasite populations, and the consequent under-detection of low-density infections that are detectable only through PCR-based molecular surveillance (6, 7). Furthermore, asymptomatic and sub-patent mixed-species infections involving *Plasmodium ovale* and *Plasmodium malariae* significantly undermine malaria elimination efforts, particularly among pregnant women in endemic settings, by constituting molecularly cryptic parasite reservoirs that escape detection by routine microscopy- and RDT-based surveillance, thereby sustaining low-level transmission (8, 9).

Malaria in pregnancy (MiP) is of particular concern as pregnancy-associated immunomodulation facilitates parasite persistence and placental sequestration, enabling infected women to function as reservoirs of transmission while simultaneously predisposing to adverse maternal and foetal outcomes, including maternal anemia, placental malaria, intrauterine growth restriction, low birth weight, and increased perinatal morbidity (10, 11).

Against this backdrop, the present study assesses the prevalence and seasonal dynamics of asymptomatic/sub-patent malaria infections and anemia among pregnant women in high-transmission, hard-to-reach areas of Odisha, to generate evidence to enhance surveillance and optimize risk-stratified interventions under the Durgama Anchalare Malaria Nirakaran (DAMaN) initiative, and support the National Framework for Malaria Elimination (NFME) for decision-making for this highly vulnerable and epidemiologically critical population.

## Methods

### Ethical approval

The study was approved by the Institutional Human Ethics Committee of the ICMR–Regional Medical Research Centre (Approval No. ICMR-RMRC/IHEC-2019/012; dated 27 February 2019) and by the State Research and Ethics Committee, Department of Health and Family Welfare, Government of Odisha (Approval No. 453/SHRMU187/17; dated 22 August 2017). Informed consent was obtained from all participants prior to enrolment, after they had been provided with a detailed explanation of the study proposal.

### Study setting, design, and participants

Odisha is physiographically divided into four distinct regions: the Northern Plateau, Central Tableland, Eastern Ghats, and Coastal Belt. Malaria transmission intensity varies substantially across these regions, with districts located in the Eastern Ghats and Northern Plateau exhibiting the highest malaria burden whereas the Coastal Belt has very low endemicity. To evaluate the coverage, implementation, and impact of the DAMaN program, a series of community-based assessment surveys called *DAMaN Assessment* were undertaken in selected program areas. Now, this parent *DAMaN Assessment* Project employed a repeated cross-sectional survey design using a multistage cluster sampling framework and was conducted during three survey rounds in 2019, 2021, and 2024.

For the 2019 and 2021 survey rounds, the sample size was calculated assuming a malaria parasitaemia prevalence of 3% among pregnant/lactating women, as this represented the rarest outcome of interest in the survey, and assuming that pregnant women constituted approximately 1.5–2% of the study population. An absolute precision of 1%, a 95% confidence level, and a design effect of 2 were used, yielding a target sample of 2,228 households. For the 2024 survey round, the sample size of 738 was estimated using the malaria prevalence observed during the 2021 survey round (4%) as the expected prevalence, with an absolute precision of 2%, a 95% confidence level, and a design effect of 2. For the 2019 and 2021 survey rounds, six districts were selected based on physiographic region and annual parasite incidence (API), and two blocks were selected from each district. These included Chandrapur and Kashipur blocks of Rayagada district, Biswanathpur and M. Rampur blocks of Kalahandi district, G. Udayagiri and Tikabali blocks of Kandhamal district in the Eastern Ghats region; Gurundia and Lahunipada blocks of Sundargarh district and Harichandanpur and Telkoi blocks of Keonjhar district in the Northern Plateau region; and Pallahada and Chhendipada blocks of Angul district in the Central Tableland region. Within each block, eight sub-centers were selected. From each sub-center, 24 households with at least one pregnant woman, lactating woman, or child aged <5 years were enrolled, provided the household members gave written informed consent to participate. For the 2024 survey round, the two highest malaria burden districts, Rayagada and Kalahandi, were selected. Two blocks were selected from each district (Chandrapur & Bissamcuttack from Rayagada; Lanjigarh and M. Rampur from Kalahandi), followed by the selection of 23 villages within each block. These 23 villages were selected based on the intensity of DAMaN Programme (category 1: villages with intensive DAMaN; category 2: villages with intermediate; and category 3: least rounds of DAMaN villages). From each village, 9 households meeting the same eligibility criteria were enrolled after obtaining written informed consent.

**Figure 1:**
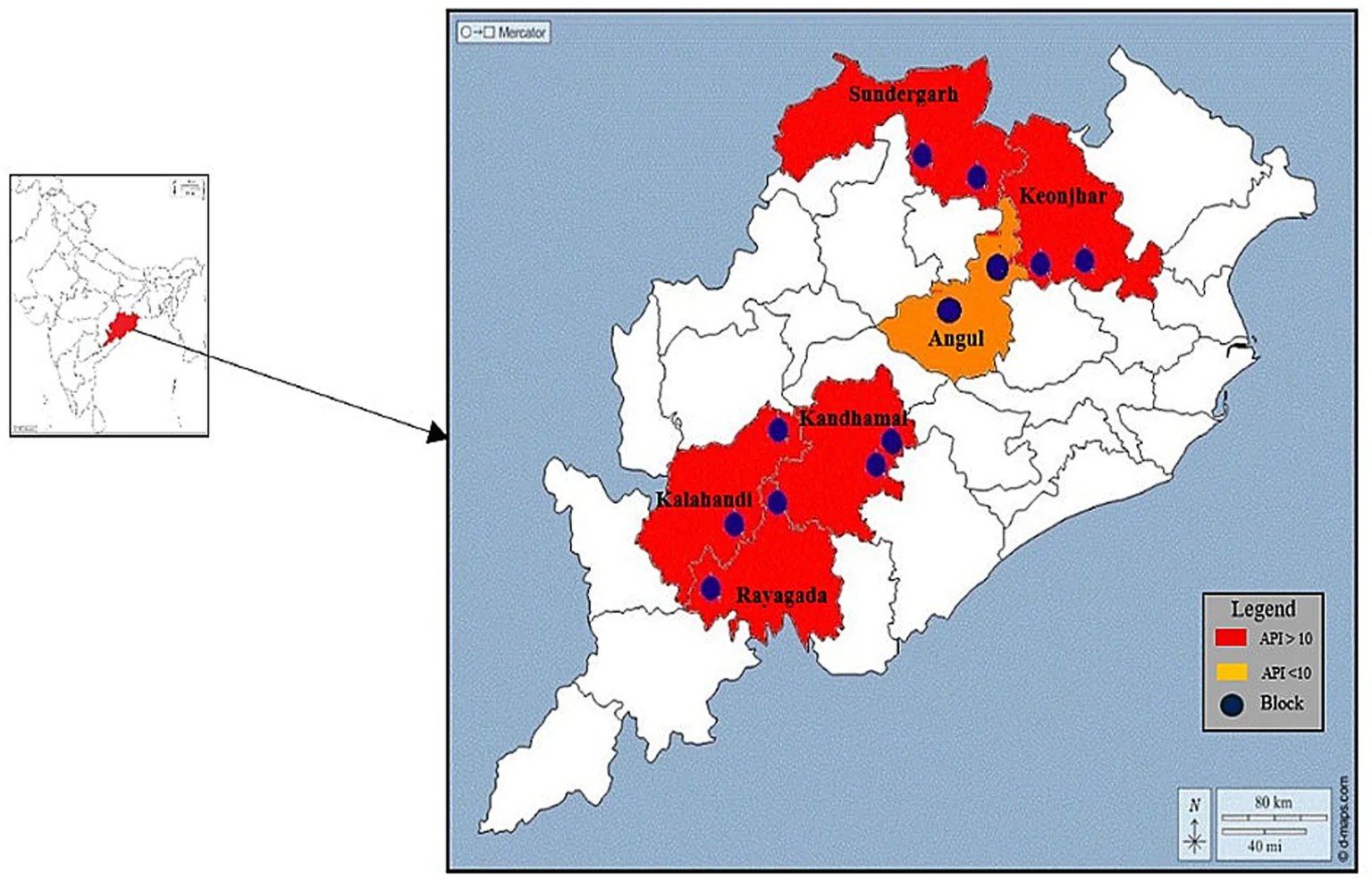
Map of Odisha highlights the Annual Parasite Index (API) status of six districts of the year NVBDCP, 2015 (13) and studied blocks in the present study. The base maps were taken from the website https://www.d-maps.com/carte.php?num_car=272262&lang=en.

Across the three survey rounds, 1,823, 1,971, and 928 households were surveyed in 2019, 2021, and 2024, respectively, resulting in the coverage of 8,477, 8,900, and 3,841 individuals. Of these, 3,557 (42.0%), 4,236 (47.6%), and 2,922 (76.1%) participants, respectively, provided blood samples for malaria diagnosis and hemoglobin estimation. The geographical distribution of the study sites is shown in Figure 1 (12).

The present study included all pregnant women enrolled during the parent *DAMaN Assessment* project. All pregnant women residing in sampled households and available at the time of the survey were invited to participate irrespective of gravida or gestational age. Women were eligible for inclusion if they were currently pregnant, residing in the study area during the survey period, provided written informed consent, and agreed to provide blood samples for malaria testing and hemoglobin estimation. A total of 350 pregnant women were enrolled during the 2019 survey, 336 during the 2021 survey, and 175 during the 2024 survey, yielding a final analytical sample of 861 pregnant women.

### Demographic data and blood analysis

Demographic information, including age and village of residence was collected from each participant. Axillary body temperature was measured using an infrared digital thermometer (Neureca, India), and an axillary temperature having ≥ 37.8 °C (100 °F) was recorded as fever. Approximately 500 μL of venous blood was aseptically collected in a BD Vacutainer® EDTA vial by a trained laboratory technician. Of the collected blood, at least 50 μL of blood was used to prepare thin and thick blood smears for microscopic examination, and 10 μL of blood was used for the Standard Q Malaria *Pf/PAN* rapid diagnostic test (SD Biosensor, India) to screen the malaria (13) in all study sites during the entire study period to maintain consistency in malaria diagnosis. Thick and thin blood smears were stained with Giemsa and examined by microscopists following standard WHO procedures.

For hemoglobin estimation by HemoCue Hb 201 + analyser (Angelholm, Sweden) finger prick blood samples were collected from each participant. Classification of anemia was done as per the WHO guidelines on hemoglobin cutoffs to define anemia in individuals and populations (14). The blood samples were transported to the ICMR-Regional Medical Research Centre, Bhubaneswar, within 24 h of collection, maintaining proper cold chain for further molecular analysis. All pregnant women who tested positive by RDT during field screening were immediately referred to the nearest health facility and treated with Artesunate-Sulfadoxine+Pyrimethamine (ASSP) for *falciparum* and Chloroquine (CQ) for other non-*falciparum* species according to the National Centre for Vector Borne Diseases Control (NCVBDC) guidelines (15). Women identified subsequently as PCR-positive but RDT-negative were communicated to the concerned district health authority for appropriate management and subsequent follow-up.

### Molecular detection of *plasmodium* species by nested PCR

Parasite genomic DNA was extracted from 200 μL of EDTA-anticoagulated whole blood using the QIAamp DNA Blood Mini Kit (QIAGEN, Hilden, Germany) according to the manufacturer’s instructions and eluted in 50 μL of TE buffer. Under optimized laboratory conditions, the nested PCR assay used in this study has an estimated detection sensitivity of approximately 1–5 parasites/μL of blood (16, 17).

Nested PCR targeting the small subunit ribosomal RNA (18S rRNA) gene was performed using species-specific primers to detect *Plasmodium falciparum*, *P. vivax*, *P. malariae*, and *P. ovale* (18)(Supplementary Table 3). Amplification was carried out in a thermal cycler (Bio-Rad T100, Hercules, CA, USA). The primary PCR was performed in a 25 μL reaction mixture containing 0.2 U Taq DNA polymerase, 0.2 mM of each dNTP, 0.4 μM of each primer, 2.0 mM MgCl₂, and the appropriate reaction buffer (3B BlackBio Biotech India Ltd., India). Cycling conditions consisted of an initial denaturation at 94 °C for 1 min, followed by 35 cycles of denaturation at 94 °C for 1 min, annealing at 50 °C for 1 min, and extension at 72 °C for 1 min, with a final extension at 72 °C for 10 min.

The nested PCR was conducted under identical reaction conditions, except that the annealing temperature was increased to 55 °C. Amplified products were separated by electrophoresis on a 2.0% (w/v) agarose gel stained with ethidium bromide and visualized under UV illumination. Species identification was based on comparison with a 100-bp DNA ladder (3B BlackBio Biotech India Ltd., India) and expected amplicon sizes: *P. vivax* (120 bp), *P. malariae* (144 bp), *P. falciparum* (205 bp), and *P. ovale* (approximately 800 bp). *Plasmodium knowlesi* was not investigated in the present study. Gel images were captured using a gel documentation system (iGene Labserve Pvt. Ltd., India). Previously confirmed species-specific *Plasmodium* DNA samples were included as positive controls, while genomic DNA extracted from malaria-negative individuals served as negative controls.

Differentiation of the two *P. ovale* subspecies, *P. ovale curtisi* and *P. ovale wallikeri*, was performed by amplification of a partial fragment of the 18S rRNA gene using a two-step PCR approach, as described in Bal et al. (8) (Supplementary Table 3).

Each PCR reaction was carried out in a final volume of 25 μL containing 0.2 U Taq DNA polymerase, 0.2 mM of each dNTP, 0.4 μM of each primer, and 2.0 mM MgCl₂ (3B BlackBio Biotech India Ltd., India). The primary PCR cycling conditions consisted of an initial denaturation at 94 °C for 1 min, followed by 35 cycles of denaturation at 94 °C for 1 min, annealing at 50 °C for 1 min, and extension at 72 °C for 1 min, with a final extension at 72 °C for 10 min. The nested PCR was performed under identical conditions, except that the annealing temperature was increased to 55 °C. Amplified products were separated by electrophoresis on a 2.0% (w/v) agarose gel stained with ethidium bromide and visualized under UV illumination. Species identification was based on comparison with a 100-bp DNA ladder (3B BlackBio Biotech India Ltd., India) and expected amplicon sizes: *P. o. wallikeri* (938 bp), *P. o. curtisi* (827 bp).

### Statistical analysis

Data were analyzed using R software version 4.4.0 (A Language and Environment for Statistical Computing_. R Foundation for Statistical Computing, Vienna, Austria). Participant characteristics were summarised using descriptive statistics. Continuous variables were presented as median and interquartile range (IQR), while categorical variables were summarised as frequencies and percentages. The prevalence of malaria infection, anemia, and sub-patent malaria infection was estimated overall and by survey phase (2019, 2021, and 2024), with corresponding 95% confidence intervals (CIs) calculated using the exact binomial method. Differences in participant characteristics according to malaria infection, anemia, and sub-patent malaria status were assessed using Pearson’s chi-square test or Fisher’s exact test, as appropriate.

Multivariable logistic regression models were fitted to identify factors associated with (i) malaria infection (malaria-positive versus malaria-negative), (ii) sub-patent malaria infection among malaria-positive women (sub-patent versus patent infection). Covariates were selected *a priori* based on biological plausibility and existing evidence and included transmission setting, maternal age, gravidity, trimester of pregnancy, indoor residual spraying (IRS), insecticide-treated net (ITN) ownership and use, symptomatic status, and malaria infection status, as appropriate for each model. Survey year was not included because it was highly correlated with transmission setting and was considered a proxy measure of underlying transmission intensity. All multivariable logistic regression models were estimated using cluster-robust standard errors to account for clustering.

For the purpose of this study, asymptomatic malaria was defined as the presence of malaria infection in the absence of fever or other malaria-related clinical symptoms at the time of screening, irrespective of the diagnostic method used. Patent malaria was defined as malaria infection detected by conventional diagnostic methods, namely microscopy and/or rapid diagnostic test (RDT). Sub-patent malaria was defined as malaria infection that was negative by both microscopy and RDT but positive by nested polymerase chain reaction (PCR), indicating parasite densities below the detection threshold of conventional diagnostic methods.

The association between malaria infection and anemia during pregnancy was further examined using univariable and multivariable logistic regression. The adjusted model controlled for transmission setting, maternal age, gravidity, trimester of pregnancy, household bed-net ownership, bed-net use and Indoor residual spraying (IRS). Results are presented as odds ratios (ORs) or adjusted odds ratios (aORs) with 95% confidence intervals (CIs).

## Results

A total of 861 pregnant women participated in the survey conducted between 2019 and 2024 across 12 blocks in six malaria-endemic districts of Odisha. The median age of participants was 25 years (IQR 7 years), and the largest proportion belonged to the 21–25-year age group (37.7%). Most participants were daily wage laborers (58.0%), had no formal education (42.9%), and were in the second trimester of pregnancy (48.7%). Primigravidae constituted 36.8% of the study population. Ownership of insecticide-treated nets (ITNs) was high (91.4%), while 80.0% reported sleeping under an ITN and having received indoor residual spraying (IRS) within the preceding 12 months (Table 1).

**Table 1:** Socio-demographic, obstetric, and malaria-prevention characteristics of enrolled pregnant women (*N* = 861).

Characteristic	Overall (*n* = 861) *n* (%)
Age group (years)	
Median (IQR)	25 (7)
18–20	167 (19.4)
21–25	325 (37.7)
26–30	236 (27.4)
31–35	89 (10.3)
36–40	31 (3.6)
41–45	13 (1.5)
Educational status	
No formal education	369 (42.9)
Primary school	242 (28.1)
Secondary school	168 (19.5)
College/University	82 (9.5)
Occupation	
Daily wage laborer	499 (58.0)
Farmer	310 (36.0)
Housewife	52 (6.0)
Trimester	
First trimester	173 (20.1)
Second trimester	419 (48.7)
Third trimester	269 (31.2)
Gravida	
Primigravidae	317 (36.8)
Secundigravidae	255 (29.6)
Multigravidae	289 (33.6)
Insecticide-treated net (ITN) ownership	
Yes	787 (91.4)
No	74 (8.6)
Insecticide-treated net (ITN) use	
Yes	689 (80.0)
No	172 (20.0)
Indoor residual spraying (IRS)	
Yes	689 (80.0)
No	172 (20.0)

Of the 861 enrolled pregnant women, 350 were recruited during the high-transmission season in 2019, 336 during the low-transmission season in 2021, and 175 during the high-transmission season in 2024. Overall, 284 women (33.0%; 95% CI: 29.9–36.3) were classified as malaria positive based on RDT, microscopy and/or PCR results, while 577 (67.0%) were malaria negative. Moreover, species wise prevalence of malaria out of 269 PCR popsitive in 2019, 2021 and 2024 revealed that single species infection (mono-infection) was 84.3% (151 out of 179), 92.6% (25 out of 27) and 60.3% (38 out of 63), while mixed infections were 15.6% (28 out of 179), 7.4% (2 out of 27) and 39.6% (25 out of 63) respectively. Among the mono-infection, *Plasmodium vivax* was the most predominant species in 2019 (77 out of 179; 43.0%) and 2024 (18 out of 63; 28.5%). However, in 2019 *P. malariae* was found to be second most prevalent species (56 out of 179; 31.2%) followed by *P. falciparum* in 17 cases out of 179 (9.5%), and *P. ovale* in 1 case out of 179 (0.5%) in contrast to 2024 where *P. falciparum* (17 out of 63; 27%) was the second most prevalent species followed by *P. ovale* (3 out of 63; 4.7%). With respect to mixed infections, *P. falciparum* plus *P. vivax* (*Pf*+*Pv*) was the most common combination, accounting for 18 cases out of 179 (10.0%), followed by *Pf*+*Pv*+*Pm* in 6 cases out of 179 (3.3%), and *Pv*+*Pm* and *Pf*+*Pm* in 2 cases each out of 179 (0.5%) in 2019, while *Pf*+*Po* was detected in 10 cases out of 63 (15.8%), *Pf*+*Pv* in 3 cases out of 63 (4.7%), *Pv*+*Po* in 1case out of 63 (1.6%) and *Pf*+*Pv*+*Po* in 10 cases out of 63 (15.8%) and *Pf*+*Pm*+*Po* in 1 case out of 63 (1.6%) in 2024. But in 2021 *P. falciparum* was found to be most prevalent (16 out of 27; 59.2%) single species infection followed by *P. vivax* in 4 cases out of 27 (14.8%) and *P. malariae* in 5 cases out of 27 (18.5%). Whereas mixed infections were found only in two cases out of 27 (7.4%) i.e. *Pf*+*Pm* in one case and *Pf*+*Pv* in one case (Supplementary Table 1).

**Figure 2:**
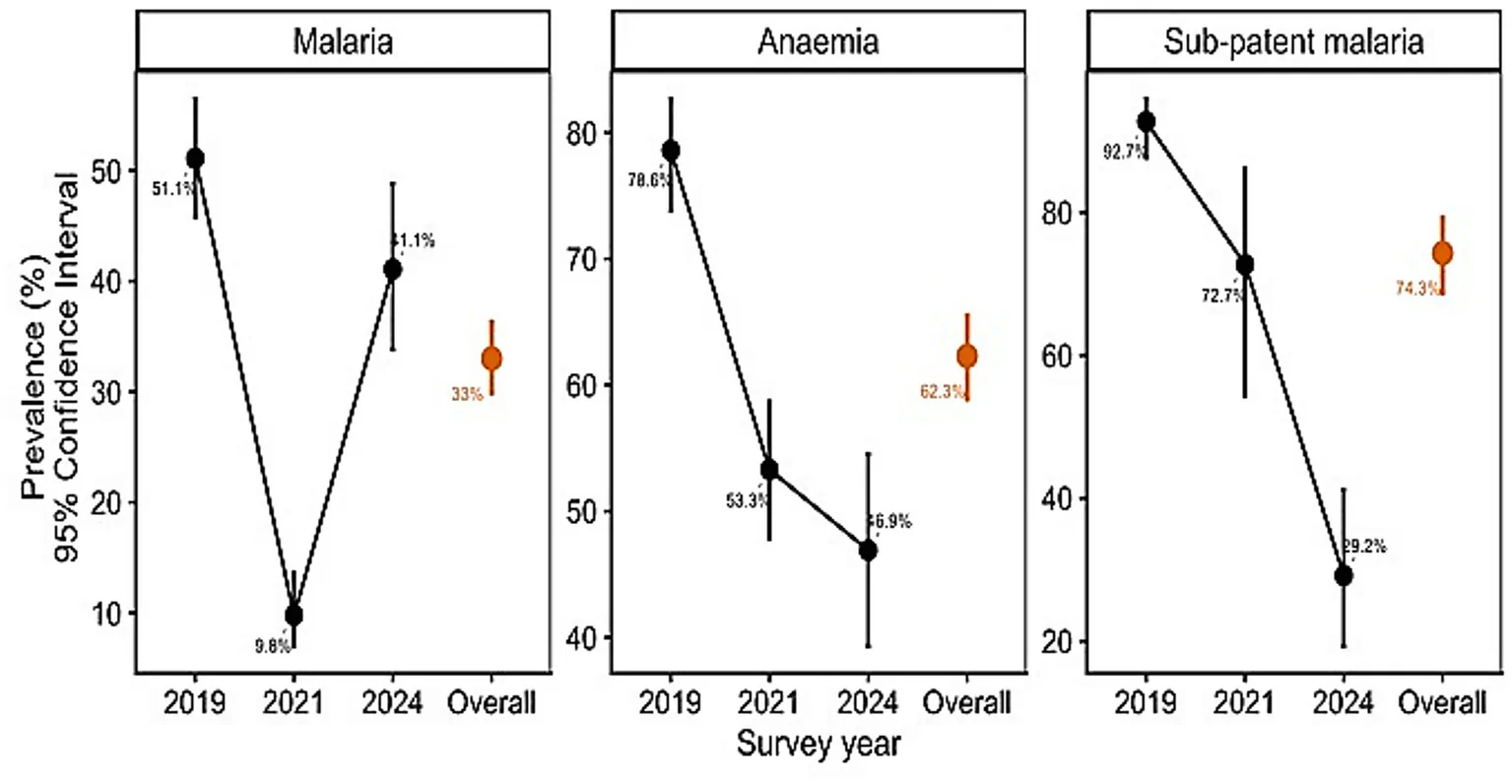
Prevalence of malaria, anemia, and sub-patent malaria among pregnant women by survey year, Odisha, India (2019–2024).

**Figure 3:**
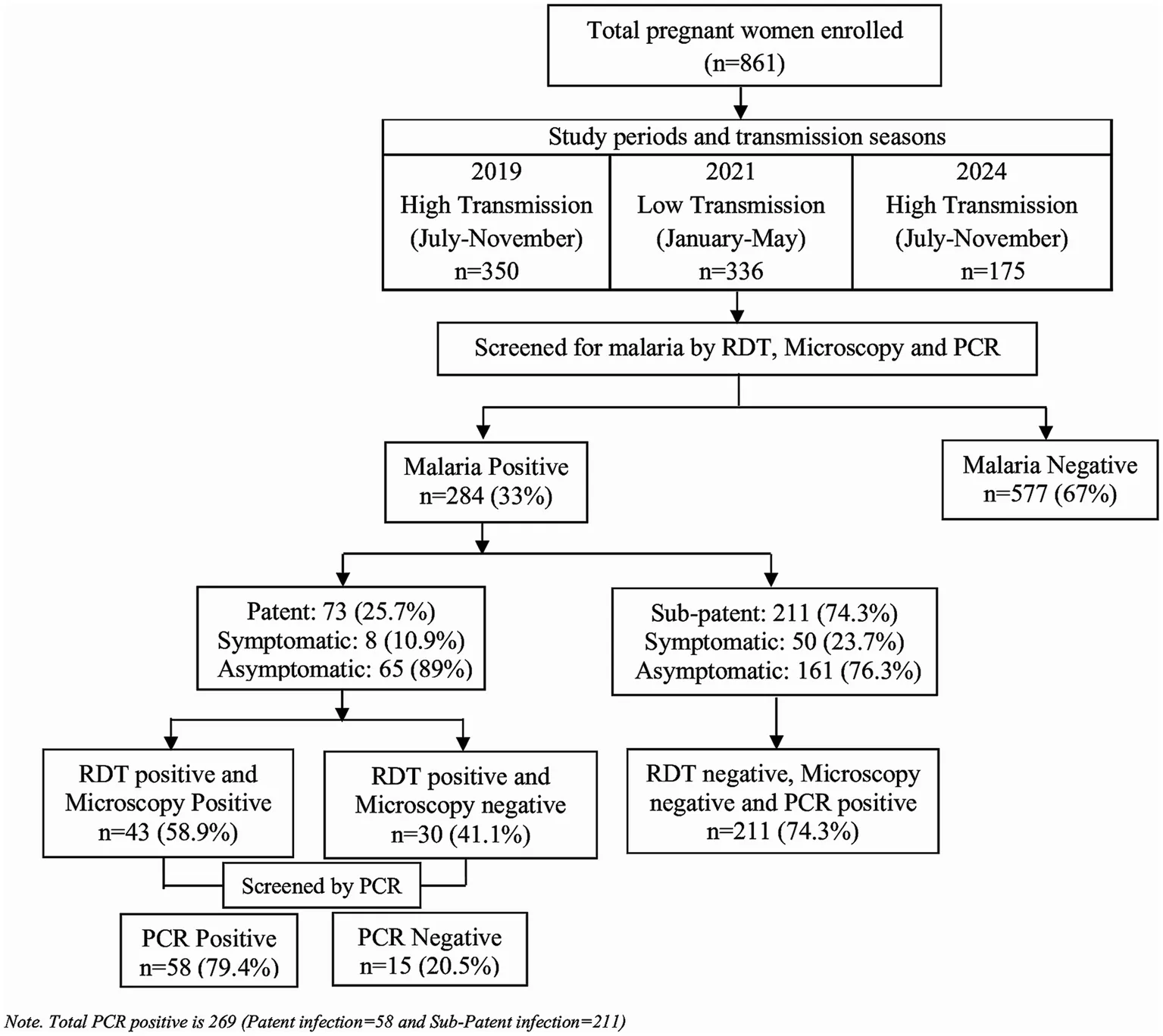
Study population and diagnostic approach used to classify malaria infections among pregnant women.

Malaria prevalence varied substantially across survey phases, declining from 51.1% (95% CI: 45.8–56.5) in 2019 to 9.8% (95% CI: 7.0–13.6) in 2021 before increasing to 41.1% (95% CI: 33.8–48.8) in 2024 (Figure 2). Among the 284 malaria-positive women, 58 (20.4%) were RDT positive/PCR positive, 211 (74.3%) had sub-patent malaria (RDT-negative and microscopy-negative but PCR-positive), and 15 (5.3%) were RDT-positive/PCR-negative. Overall, 79.6% malaria-positive women were asymptomatic, whereas 58 (20.4%) reported malaria-related symptoms. Among asymptomatic malaria cases, 71.2% were classified as sub-patent infections. The diagnostic classification of study participants is shown in Figure 3.

Participant characteristics differed significantly across malaria status. Malaria prevalence was significantly higher during the high-transmission season than during the low-transmission season (47.8% vs. 9.8%, *p* < 0.001). Among malaria-positive pregnant women, the distribution of cases was 88.4% during the high-transmission season and 11.6% during the low-transmission season. Likewise, among malaria-positive women, 79.6% were asymptomatic and 20.4% were symptomatic (*p* < 0.001) (Table 2). Age was also associated with malaria infection (*p* = 0.026), whereas gravidity and trimester were not significantly associated. In multivariable analysis, residence in a high-transmission season has 8 times higher odds of malaria infection compared with those living in low-transmission season (aOR: 8.23; 95% CI: 4.30–15.78). Indoor residual spraying (IRS) (aOR: 0.02; 95% CI: 0.01–0.04) and bed-net use (aOR: 0.25; 95% CI: 0.10–0.62) were independently associated with lower odds of malaria infection. Maternal age, gravidity, trimester of pregnancy, and household bed-net ownership were not significantly associated with malaria infection (Table 3).

**Table 2:** Comparison of participant characteristics according to malaria infection (RDT, microscopy, nest-PCR positive) status among enrolled pregnant women (*N* = 861).

Characteristic	Malaria negative (*n* = 577) (*n*%)	Malaria positive (*n* = 284) (*n*%)	*p*-value
Age group (years)			0.026
18–20	109 (18.9)	58 (20.4)	
21–25	219 (38.0)	106 (37.3)	
26–30	171 (29.6)	65 (22.9)	
31–35	55 (9.5)	34 (12.0)	
36–40	19 (3.3)	12 (4.2)	
41–45	4 (0.7)	9 (3.2)	
Transmission season			<0.001
Low	303 (52.5)	33 (11.6)	
High	274 (47.5)	251 (88.4)	
Symptomatic status			<0.001
Symptomatic	61 (10.6)	58 (20.4)	
Asymptomatic	516 (89.4)	226 (79.6)	
Gravida			0.762
Primigravidae	216 (37.4)	101 (35.6)	
Secundigravidae	172 (29.8)	83 (29.2)	
Multigravidae	189 (32.8)	100 (35.2)	
Trimester			0.515
First	119 (20.6)	54 (19.0)	
Second	285 (49.4)	134 (47.2)	
Third	173 (30.0)	96 (33.8)	
Household bed-net ownership			<0.001
No	34 (5.9)	40 (14.1)	
Yes	543 (94.1)	244 (85.9)	
Bed-net use			<0.001
No	64 (11.1)	108 (38.0)	
Yes	513 (88.9)	176 (62.0)	
Indoor residual spraying (IRS)			<0.001
No	11 (1.9)	161 (56.7)	
Yes	566 (98.1)	123 (43.3)	

**Table 3:** Factors associated with malaria infection (RDT, microscopy, nest-PCR positive) among pregnant women: Multivariable logistic regression analysis (*n* = 861).

Variable	Crude OR (95%CI)	*p*-value	Adjusted OR (95% CI)	*p*-value
Transmission seasons				
Low			Reference	
High	8.41 (4.83–14.65)	<0.0001	8.23 (4.30–15.78)	<0.0001
Maternal age (years)	1.02 (0.99–1.05)	0.097	1.02 (0.97–1.07)	0.482
Gravida	1.06 (0.91–1.25)	0.443	1.09 (0.80–1.40)	0.599
Trimester	1.12 (0.91–1.37)	0.292	1.19 (0.91–1.56)	0.209
Household bed-net ownership				
No	Reference		Reference	
Yes	0.38 (0.22–0.66)	0.001	1.80 (0.61–5.30)	0.284
Bed-net use				
No	Reference		Reference	
Yes	0.20 (0.13–0.33)	<0.0001	0.25 (0.10–0.62)	0.003
Indoor residual spraying (IRS)				
No	Reference		Reference	
Yes	0.02 (0.01–0.03)	<0.0001	0.02 (0.01–0.04)	<0.0001

Maternal age was modeled as a continuous variable. Gravidity and trimester were modeled as ordinal (numeric) variables representing increasing gravida and advancing gestational age, respectively. Odds ratios for gravidity and trimester represent the change in odds associated with a one-category increase.

The overall prevalence of sub-patent malaria was reported to be 74.3% (95% CI: 68.7–79.2) among the pregnant mothers infected with malaria. It varied significantly across household bed net ownership (*p* < 0.001) and bed-net use (*p* < 0.001) (Table 4). However, in multivariable logistic regression analysis restricted to malaria-positive women, increasing maternal age was associated with higher odds of sub-patent malaria (aOR: 1.05; 95% CI: 1.01–1.15). Higher gravidity was also independently associated with sub-patent infection (aOR: 1.70; 95% CI: 1.07–1.25). Women reporting bed-net use had substantially higher odds of sub-patent malaria than patent malaria (aOR: 6.99; 95% CI: 2.68–18.24), and those residing in households that had received Indoor residual spraying (IRS) had higher odds of sub-patent infection (aOR: 2.10; 95% CI: 1.35–5.14). Transmission season, trimester of pregnancy, and anemia were not significantly associated with sub-patent malaria (Table 5).

**Table 4:** Comparison of participant characteristics according to sub-patent infection status among malaria-positive pregnant women (*n* = 284).

Characteristic	Sub-patent (*N* = 211) (*n*%)	Patent (*N* = 73) (*n*%)	*p*-value
Age group (years)			0.004
18–20	32 (15.2)	26 (35.6)	
21–25	86 (40.8)	20 (27.4)	
26–30	48 (22.7)	17 (23.3)	
31–35	30 (14.2)	4 (5.5)	
36–40	9 (4.3)	3 (4.1)	
41–45	6 (2.8)	3 (4.1)	
Transmission season			0.826
High	187 (88.6)	64 (87.7)	
Low	24 (11.4)	9 (12.3)	
Symptomatic status			0.020
Asymptomatic	161 (76.3)	65 (89.0)	
Symptomatic	50 (23.7)	8 (11.0)	
Gravida			0.068
Primigravidae	69 (32.7)	32 (43.8)	
Secundigravidae	69 (32.7)	14 (19.2)	
Multigravidae	73 (34.6)	27 (37.0)	
Trimester			0.405
First trimester	39 (18.5)	15 (20.5)	
Second trimester	96 (45.5)	38 (52.1)	
Third trimester	76 (36.0)	20 (27.4)	
Household bed-net ownership			<0.001
No	15 (7.1)	25 (34.2)	
Yes	196 (92.9)	48 (65.8)	
Bed-net use			<0.001
No	53 (25.1)	55 (75.3)	
Yes	158 (74.9)	18 (24.7)	
Indoor residual spraying (IRS)			0.037
No	112 (53.1)	49 (67.1)	
Yes	99 (46.9)	24 (32.9)	

**Table 5:** Factors associated with sub-patent malaria among malaria-positive pregnant women: multivariable logistic regression analysis (*n* = 284).

Variable	Crude OR (95% CI)	*p*-value	Adjusted OR (95% CI)	*p*-value
Transmission season				
Low	Reference		Reference	
High	1.10 (0.38–3.15)	0.865	0.87 (0.19–4.05)	0.863
Maternal age (years)	1.05 (1.00–1.12)	0.04	1.05 (1.01–1.15)	0.03
Gravida	1.13 (1.05–1.55)	0.001	1.70 (1.07–1.25)	0.007
Trimester	1.23 (0.80–1.90)	0.342	1.17 (0.67–2.02)	0.580
Household bed-net ownership				
No	Reference		Reference	
Yes	6.81 (2.83–16.37)	<0.001	2.06 (0.72–5.86)	0.177
Bed-net use				
No	Reference		Reference	
Yes	9.11 (3.97–20.91)	<0.001	6.99 (2.68–18.24)	<0.001
Indoor residual spraying (IRS)				
No	Reference		Reference	
Yes	1.81 (1.04–3.49)	0.005	2.10 (1.35–5.14)	<0.001

Maternal age was modeled as a continuous variable. Gravidity and trimester were modeled as ordinal (numeric) variables representing increasing gravida and advancing gestational age, respectively. Odds ratios for gravidity and trimester represent the change in odds associated with a one-category increase.

Overall, 62.3% (95% CI: 58.9–65.5) pregnant women were anaemic. Anemia prevalence was highest during Phase 1 in 2019 (78.6%; 95% CI: 73.8–82.7), followed by Phase 2 in 2021 (53.3%; 95% CI: 47.8–58.7) and Phase 3 in 2024 (46.9%; 95% CI: 39.3–54.5) (Figure 2). Anemia prevalence varied significantly according to transmission season, survey phase, and symptomatic status (all *p* < 0.001) (Table 6).

**Table 6:** Comparison of participant characteristics according to anemia status among enrolled pregnant women (*N* = 861).

Characteristic	No anemia (*n* = 325) (*n*%)	Anemia (*n* = 536) (*n*%)	*p*-value
Age group (years)			0.243
18–20	53 (16.3)	114 (21.3)	
21–25	132 (40.6)	193 (36.0)	
26–30	88 (27.1)	148 (27.6)	
31–35	39 (12.0)	50 (9.3)	
36–40	10 (3.1)	21 (3.9)	
41–45	3 (0.9)	10 (1.9)	
Malaria status			0.220
Negative	226 (69.5)	351 (65.5)	
Positive	99 (30.5)	185 (34.5)	
Sub-patent infection			0.158
No	254 (78.2)	396 (73.9)	
Yes	71 (21.8)	140 (26.1)	
Transmission season			<0.001
High	168 (51.7)	357 (66.6)	
Low	157 (48.3)	179 (33.4)	
Symptomatic status			<0.001
Asymptomatic	298 (91.7)	444 (82.8)	
Symptomatic	27 (8.3)	92 (17.2)	
Plasmodium species			0.327
Negative	231 (71.1)	361 (67.4)	
*P. falciparum*	23 (7.1)	27 (5.0)	
*P. malariae*	20 (6.2)	41 (7.6)	
*P. ovale*	1 (0.3)	3 (0.6)	
*P. vivax*	29 (8.9)	70 (13.1)	
Mixed infection	21 (6.5)	34 (6.3)	
Gravida			0.051
Primigravidae	110 (33.8)	207 (38.6)	
Secundigravidae	112 (34.5)	143 (26.7)	
Multigravidae	103 (31.7)	186 (34.7)	
Trimester			0.496
First	72 (22.2)	101 (18.8)	
Second	155 (47.7)	264 (49.3)	
Third	98 (30.2)	171 (31.9)	
Household bed-net ownership			0.023
No	37 (11.4)	37 (6.9)	
Yes	288 (88.6)	499 (93.1)	
Bed-net use			0.003
No	82 (25.2)	90 (16.8)	
Yes	243 (74.8)	446 (83.2)	
Indoor residual spraying (IRS)			0.223
No	58 (17.8)	114 (21.3)	
Yes	267 (82.2)	422 (78.7)	

Among malaria-positive women, 185 of 284 (65.1%) were anaemic compared with 351 of 577 (60.8%) malaria-negative women. However, logistic regression analysis showed no significant association between malaria infection and anemia. The crude odds ratio for anemia among malaria-positive women was 1.20 (95% CI: 0.81–1.78), which remained non-significant after adjustment for transmission season, maternal age, gravidity, and trimester of pregnancy (aOR: 0.98; 95% CI: 0.55–1.74) (Supplementary Table 2).

## Discussion

The present community-based study, conducted at three time points between 2019 and 2024 among pregnant women residing in high-burden, malaria-endemic, hard-to-reach tribal districts of Odisha, demonstrates high prevalence of asymptomatic (79.6%) and sub-patent (74.3%) malaria infections. Furthermore, the study demonstrates seasonal variation in infection pattern, circulation of mixed *Plasmodium* species infection and no association between anemia and malaria infection in the studied pregnant women.

As the primary objective of this study was to assess the burden of asymptomatic and sub-patent malaria infections in hard-to-reach, malaria-endemic regions of Odisha characterized by persistent transmission, a comprehensive diagnostic approach was adopted for malaria screening. All study participants, including both suspected febrile cases and individuals without any clinical signs or symptoms suggestive of malaria, were screened using microscopy, RDT and PCR assay. The inclusion of multiple diagnostic modalities enabled improved detection of low-density and asymptomatic infections that are frequently missed by conventional diagnostic methods, thereby providing a more accurate estimate of the hidden parasite reservoir contributing to sustained malaria transmission in these underserved populations. Comparison of diagnostic modalities in the present study demonstrated that microscopy could detect malaria only in 15.1% (43 out of 284) cases particularly among malaria suspected febrile cases. This finding highlights the limited sensitivity of conventional microscopy in detecting low-density and sub-patent infections during pregnancy, where parasite sequestration and reduced peripheral parasitaemia are common phenomena. In contrast, PCR identified 74.3% (211 out of 284) a substantially higher number of infections, including mixed-species infections involving *P. falciparum, P. vivax, P. malariae,* and *P. ovale*, many of which would have remained undetected under routine surveillance (6). Similar observations have been reported from malaria-endemic regions in Southeast Asia including India, and Africa, where molecular methods revealed a considerable hidden reservoir of submicroscopic infections among pregnant women (6, 19). Although microscopy and RDTs remain operationally feasible and cost-effective tools for field-level diagnosis, their reduced sensitivity in low-density infections may underestimate the true burden of malaria in pregnancy and allow persistent parasite reservoirs to sustain transmission. Although PCR-based molecular diagnostics provide substantially higher sensitivity for detecting low-density and mixed-species infections, their routine implementation in peripheral public health settings remains constrained by cost, infrastructure requirements, technical expertise, and turnaround time. Therefore, targeted molecular surveillance in sentinel high-burden areas may represent a more pragmatic approach.

Substantially high prevalence of malaria including asymptomatic/sub-patent infection during the monsoon/post-monsoon seasons (high-transmission) in 2019 and 2024 and low prevalence of malaria as well as asymptomatic/sub-patent infection during the low-transmission season in 2021 is at par with the observations made in other malaria endemic areas of West Africa, and across India (9, 20). The observed cyclical pattern confirms that malaria transmission peaks during and after the rainy season. This is because increased rainfall between July and November creates more breeding sites for *Anopheles* mosquitoes, leading to higher transmission rates in India and other parts of the world (21–26). However significantly high prevalence of asymptomatic cases (79.6%) in both high transmission and low transmission season among the pregnant women in our study population is an important observation. Odisha is known for high malaria endemicity since long in India, contributing majority of the total malaria cases to the national pool (27). It has been shown that individuals living in endemic areas develops a form of naturally acquired, partial, and non-sterilizing immunity, called premunition, to malaria after repeated exposure to the plasmodium infection (28). This state of immunity allows individuals, particularly adults in high-transmission areas, to maintain low-density, often subpatent (undetectable by microscopy) infections without displaying clinical symptoms, thereby serving as asymptomatic reservoirs. This might be the cause of high prevalence of asymptomatic /subpatent cases in our populations which was not reported earlier because of non-availability of molecular tools for diagnosis. These low-density infections persist longer in the host during dry seasons might be because of less competition between parasite strains, and reduced host immune activation (29, 30). Similar observations have also been made in Bandiagara region Central Mali, West Africa (31), Eastern Sudan (32), School-age Children as Asymptomatic Malaria Reservoir in Tribal Villages of Bastar Region, Chhattisgarh (33). However, in the present study the higher proportion of symptomatic infections among RDT-negative/PCR-positive women as compared to RDT positive may be due to pregnancy-associated immune modulation that alter host–parasite interactions and clinical presentation (34). Further, many symptomatic women may have been in the early stage of infection or harboring parasite densities below the detection threshold of RDTs but detectable by PCR. Another important explanation for discordant RDT and PCR results might be the limitation of HRP2-based RDTs, including low antigen concentration, variability in antigen clearance, and *pfhrp2/pfhrp3* gene deletions, which can produce false-negative RDT results despite PCR-confirmed infection (35, 36). Similar findings have been reported in Odisha (37), and other parts of Central India, where a substantial proportion of malaria infections during pregnancy were detected only by PCR, i.e., 66.3% of infections identified during antenatal screening were sub microscopic (38), while in our own study, we observed that 50.3% of pregnant women harbored PCR-detectable infections that were not identified by routine diagnostic methods (6). Collectively, these factors may explain the unexpectedly higher proportion of symptomatic infections among PCR-positive/RDT-negative women in malaria-endemic settings.

The present study found that ITN use and IRS were associated with higher odds of sub-patent malaria among infected pregnant women. Although seemingly paradoxical, this does not imply that these interventions increase malaria risk. Rather, ITNs and IRS may reduce parasite exposure and suppress parasite densities, resulting in infections that remain below the detection threshold of microscopy and RDTs and are detectable only by molecular methods. This explanation is supported by evidence that sub-patent infections are predominantly low-density infections and become increasingly common as transmission declines and malaria control measures intensify (39). These findings suggest that while vector-control interventions such as ITNs and IRS remain essential for reducing malaria transmission, they may be insufficient on their own to eliminate the substantial reservoir of sub-patent infections. This underscores the need for sensitive point-of-care molecular diagnostic tools capable of detecting low-density infections that are missed by conventional diagnostic methods, particularly in malaria elimination settings. The findings of the present study are in agreement with those reported from eastern Sudan, where no significant association was observed between malaria infection and either gestational trimester or gravidity (40). In contrast to the present study, reports from Madhya Pradesh, Jharkhand where malaria infection was significantly associated with both gravidity and gestational trimester, with a higher prevalence among primigravidae and women in the first and second trimesters (24, 41). Although no significant association was observed between overall malaria infection and increasing maternal age and higher gravidity were associated with sub-patent infection This observation supports the hypothesis, that acquired immunity in older and multigravid women may suppress parasite densities without completely clearing infection. As evidence suggest that protective immunity to placental malaria is acquired progressively over successive pregnancies, reflecting gravida-dependent modulation of host immune responses to *Plasmodium* spp. (42, 43).

The interrelationship between anemia and poverty contributes to the persistent burden of malaria in endemic regions, disproportionately affecting children and pregnant women. Poverty creates conditions favorable to malaria transmission, while malaria causes severe anemia, which in turn reduces the body’s ability to resist infection and handle the severity of the disease (44, 45). Similarly in this population though anemia was found to affect almost 62.3% of the total pregnant women included in the study and 65.1% of malaria-infected pregnant women mostly with asymptomatic/sub-patent infection, however, this association was not significant. Nevertheless, the presence of anemia among asymptomatic malaria cases during pregnancy underscores the clinical relevance of low-density (submicroscopic) infections, which frequently escape routine diagnosis and treatment and may contribute to maternal morbidity. These cases, frequently observed in malaria-endemic regions, can lead to substantial sequestration of *Plasmodium*-infected red blood cells in the placenta consistent with reports from India (46–48) and other endemic regions in Africa and Asia, including Ethiopia, Cameroon, Nigeria, Papua New Guinea, and Ghana (49–52). This supports the findings of previous studies in other settings like Thai-Myanmer border that submicroscopic malaria infection is associated with both maternal and fetal ill health in malaria endemic areas (19).

We have observed a large number of cases harboring neglected malaria parasites (*P. ovale* and/or *P. malariae*) mixed with *P. falciparum* and/or *P. vivax*. Earlier it has been reported that the decline of *P. falciparum* and *P. vivax* incidence after intensive control program leads to the increased incidence of neglected parasites (8, 53). This might be one of the reasons for detection of large number of cases with *P. ovale* and/or *P. malariae* during the present study. Because implementation of intensive Roll Back Malaria control program since 2004 followed by DAMaN in 2017 has reduced malaria cases in the state by ~ 84%. The detection of *P. malariae* and *P. ovale*, including mixed infections, highlights the growing recognition of non-*falciparum* malaria in India following the adoption of molecular diagnostics (54). Further, appropriate surveillance tools are required for the differential diagnosis of *P. ovale* and *P. malariae* because accurate species identification has important implications for long-term case management and malaria surveillance. Similarly earlier *P. falciparum* (73.5%) was the predominant parasite circulating in Odisha followed by *P. vivax* (26.4%), but recently the trend has been reversed (5). We have also observed a heterogeneity in *Plasmodium* species distribution in our study area, where *P. vivax* was predominant during high-transmission season, while *P. falciparum* during the low-transmission period. The higher prevalence of *P. vivax* may be due to inappropriate/ complete treatment linked to hypnozoite persistence, and relapses (55). Importantly, *P. vivax* infection in pregnancy has been shown to contribute significantly to maternal anemia and adverse birth outcomes in Indian settings (56). The prevalence observed in this study significantly higher than the data reported from Ethiopia and Bangladesh (57, 58), highlighting geographic disparities and the critical role of molecular tools in identifying sub-patent infections. Further, high prevalence of *P. vivax*, mixed-species infections, and neglected malaria parasites observed in the present study highlights the need for broader multivalent malaria vaccine strategies beyond *P. falciparum* alone.

## Strength and limitations

The strengths of this study include its large community-based sample, repeated surveys conducted across multiple years and transmission settings, focus on pregnant women as a vulnerable population, and the use of molecular diagnostics that enabled detection of sub-patent infections that would have been missed by conventional methods. However, several limitations should be considered. First, the repeated cross-sectional design precludes causal inference and limits assessment of temporal relationships between risk factors and infection status. Second, placental malaria was not assessed, preventing evaluation of the relationship between peripheral and placental infections. Third, participation varied across survey phases and villages, which may have introduced selection bias and affected the generalisability of the findings. Fourth, the analysis of factors associated with sub-patent infection was restricted to malaria-infected women, resulting in a relatively small number of patent infections; therefore, these findings should be interpreted with appropriate caution. Finally, information on malaria prevention practices was self-reported and may be subject to recall or social desirability bias.

## Conclusion

This study reveals a substantial hidden burden of malaria in pregnancy in the hard-to-reach malaria-endemic regions of Odisha, characterized by a high prevalence of asymptomatic and sub-patent infections, frequent mixed *Plasmodium* species infections, and a considerable burden of anemia among pregnant women. These largely undetected infections highlight important gaps in routine surveillance and may sustain residual malaria transmission, posing a challenge to elimination efforts. The findings support the integration of sensitive molecular diagnostics into sentinel surveillance systems and the evaluation of targeted preventive strategies within programmatic frameworks for pregnant women in high-burden settings. Strengthening pregnancy-focused malaria surveillance will be essential for guiding evidence-based interventions and accelerating progress toward India’s malaria elimination goal by 2030.

## Data Availability

The original contributions presented in the study are included in the article/Supplementary material, further inquiries can be directed to the corresponding author.
