# Supplementary material for: Hidden burden of malaria in pregnancy in hard-to-reach areas of Odisha, India: challenges to elimination and the case for integrating molecular surveillance
Source: Front Public Health. 2026 Aug 18;14:1862569. doi: 10.3389/fpubh.2026.1862569 (PMC13529693; doi:10.3389/fpubh.2026.1862569)
Supplement: Supplementary file 1 [file Table_1.DOCX]

**Supplementary Table 1.**  Malaria prevalence in high and low transmission seasons among pregnant women for the years 2019, 2021, and 2024.

| **Age** | **No. of Pregnant women** | **Total PCR positive**  **N (%)** | **PCR Results** | | | | | | | | | | | |
| --- | --- | --- | --- | --- | --- | --- | --- | --- | --- | --- | --- | --- | --- | --- |
|  |  |  | ***Pf***  **N (%)** | ***Pv***  **N (%)** | ***Pm***  **N (%)** | ***Po***  **N (%)** | ***PfPv***  **N (%)** | ***PfPm* N (%)** | ***PvPm* N (%)** | ***PfPo***  **N (%)** | ***PvPo***  **N (%)** | ***PfPvPm* N (%)** | ***PfPvPo* N (%)** | ***PfPmPo* N (%)** |
| **2019** | **N=350** | **N=179(51.1)** | 17(9.5) | 77(43.0) | 56(31.2) | 1(0.5) | 18(10.0) | 2(1.1) | 2(1.1) | 0(0) | 0(0) | 6(3.3) | 0(0) | (0) |
| 18-20 | 69 | 29(42.0) | 2(6.9) | 12(41.4) | 8(27.6) | 0 | 3(10.3) | 0 | 2(6.9) | 0 | 0 | 2(6.9) | 0 | 0 |
| 21-25 | 140 | 70(50.0) | 7(10.0) | 32(45.7) | 22(31.4) | 0 | 6(8.6) | 1(1.4) | 0 | 0 | 0 | 2(2.9) | 0 | 0 |
| 26-30 | 86 | 44(51.2) | 5(11.4) | 23(52.3) | 12(27.3) | 1(2.3) | 2(4.6) | 0 | 0 | 0 | 0 | 1(2.3) | 0 | 0 |
| 31-35 | 36 | 22(61.1) | 1(4.6) | 6(27.3) | 10(45.5) | 0 | 4(18.2) | 1(4.6) | 0 | 0 | 0 | 0 | 0 | 0 |
| 36-40 | 11 | 8(72.7) | 2(25.0) | 2(25.0) | 2(25.0) | 0 | 1(12.5) | 0 | 0 | 0 | 0 | 1(12.5) | 0 | 0 |
| 41-45 | 8 | 6(75.0) | 0 | 2(33.3) | 2(33.3) | 0 | 2(33.3) | 0 | 0 | 0 | 0 | 0 | 0 | 0 |
| **2021** | **N=336** | **N=27 (8.0)** | 16(59.2) | 4(14.8) | 5(18.5) | 0(0) | 1(3.7) | 1(3.7) | 0(0) | 0(0) | 0(0) | 0(0) | 0(0) | 0(0) |
| 18-20 | 65 | 9(13.8) | 5(55.5) | 1(11.1) | 2(22.2) | 0 | 1(11.1) | 0 | 0 | 0 | 0 | 0 | 0 | 0 |
| 21-25 | 119 | 10(8.4) | 6(60.0) | 1(10.0) | 3(30.0) | 0 | 0 | 0 | 0 | 0 | 0 | 0 | 0 | 0 |
| 26-30 | 108 | 5(4.6) | 2(40.0) | 2(40.0) | 0 | 0 | 0 | 1(20.0) | 0 | 0 | 0 | 0 | 0 | 0 |
| 31-35 | 34 | 3(8.8) | 3(100) | 0 | 0 | 0 | 0 | 0 | 0 | 0 | 0 | 0 | 0 | 0 |
| 36-40 | 8 | 0 | 0 | 0 | 0 | 0 | 0 | 0 | 0 | 0 | 0 | 0 | 0 | 0 |
| 41-45 | 2 | 0 | 0 | 0 | 0 | 0 | 0 | 0 | 0 | 0 | 0 | 0 | 0 | 0 |
| **2024** | **N=175** | **N=63 (36.0)** | 17(27.0) | 18(28.6) | 0(0) | 3(4.7) | 3(4.7) | 0(0) | 0(0) | 10(15.8) | 1(1.5) | 0(0) | 10(15.8) | 1(1.6) |
| 18-20 | 33 | 15(45.5) | 1(6.7) | 4(26.7) | 0 | 1(6.7) | 0 | 0 | 0 | 5(33.3) | 0 | 0 | 4(26.7) | 0 |
| 21-25 | 66 | 22(33.3) | 7(31.9) | 7(31.9) | 0 | 1(4.5) | 2(9.1) | 0 | 0 | 1(4.5) | 1(4.5) | 0 | 3(13.7) | 0 |
| 26-30 | 42 | 12(28.6) | 4(33.3) | 2(16.7) | 0 | 0 | 0 | 0 | 0 | 4(33.3) | 0 | 0 | 2(16.7) | 0 |
| 31-35 | 19 | 9(47.4) | 1(11.1) | 4(44.4) | 0 | 1(11.1) | 1(11.1) | 0 | 0 | 0 | 0 | 0 | 1(11.1) | 1(11.1) |
| 36-40 | 12 | 4(33.3) | 3(75.0) | 1(25.0) | 0 | 0 | 0 | 0 | 0 | 0 | 0 | 0 | 0 | 0 |
| 41-45 | 3 | 1(33.3) | 1(100) | 0 | 0 | 0 | 0 | 0 | 0 | 0 | 0 | 0 | 0 | 0 |
| **Total** | 861 | 269 (31.2) | 50 (18.6) | 99(36.8) | 61(22.6) | 4(1.5) | 22(8.1) | 3(1.1) | 2(0.7) | 10(3.7) | 1(0.3) | 6(2.2) | 10(3.7) | 1(0.3) |

**Supplementary Table 2.** Association between malaria infection and anaemia among pregnant women: Multivariable logistic regression analysis (N = 861)

| **Variable** | **Crude OR (95% CI)** | **Adjusted OR (95% CI)** |
| --- | --- | --- |
| Malaria infection |  |  |
| Negative | Reference | Reference |
| Positive | 1.20 (0.81–1.78) | 0.98 (0.55–1.74) |
| Adjusted for transmission season, maternal age, gravidity, trimester of pregnancy, household bed-net ownership, bed-net use and *Indoor residual spraying (IRS)* | | |

**Supplementary Table 3. Primers used for identification of *Plasmodium* species targeting 18S genes.**

| ***Plasmodium genus/species*** | **Primer Name** | **Primer Sequences (5’-3’)** | **Product Size**  **(bp)** |
| --- | --- | --- | --- |
| *Plasmodium* genus | rPLU5  rPLU6 | 5’-CCTGTTGTTGCCTTAAACTTC-3’,  5’-TTAAAATTGTTGCAGTTAAAACG-3’ | 1100 |
| *P. falciparum* | rFAL1  rFAL2 | 5’-TTAAACTGGTTTGGGAAAACCAAATATATT-3’  5’-CACAATGAACTCAATCATGACTACCCGTC-3’ | 205 |
| *P. vivax* | rVIV1  rVIV2 | 5’-CGCTTCTAGCTTAATCCACATAACTGATAC-3’  5’-ACTTCCAAGCCGAAGCAAAGAAAGTCCTTA-3’ | 120 |
| *P. malariae* | rMAL1  rMAL2 | 5’-ATAACATAGTTGTACGTTAAGAATAACCGC-3’  5’-AAAATTCCCATGCATAAAAAATTATACAAA-3’ | 144 |
| *P. ovale* | rOVA1  rOVA | 5’-ATCTCTTTTGCTATTTTTTAGTATTGGAGA-3’  25’-GGAAAAGGACACATTAATTGTATCCTAGTG-3’ | 800 |
| *P. ovale curtisi* | PO1  PO2 | 5′-CTACTTGACATTTCTACTTACA-3′  5′-CGTTCTTGATTAATGGAAGTAT-3′ | 827 |
| *P. ovale wallikeri* | PO3  PO4 | 5′-GCTGTAGCTAATACTTGCTTTA-3′  5′-TTCACCTCTGACATCTGAATC-3′ | 938 |
